# Rapid Assessment of COVID-19 Screening Program for Travelers in Iran: A Qualitative Study

**DOI:** 10.1017/dmp.2021.219

**Published:** 2021-07-12

**Authors:** Hamed Seddighi, Ibrahim Salmani, Hossein Baharmand, Saeideh Seddighi, Mehrab Sharifi Sedeh

**Affiliations:** 1 Campus Fryslân, University of Groningen, Leeuwarden, the Netherlands; 2 Student Research Committee, University of Social Welfare and Rehabilitation Sciences, Tehran, Iran; 3 Department of Health in Disaster and Emergency, School of Public Health, Shahid Sadoughi University of Medical Sciences, Yazd, Iran; 4 Department of ICT, University of Agder, Norway; 5 Social Science Department, Tehran University, Tehran, Iran; 6 School of Public Health, Tehran University of Medical Science (TUMS), Iran

**Keywords:** humanitarian response, logistic, mental health, ethics, volunteering

## Abstract

**Objective:**

Coronavirus disease 2019 (COVID-19) screening stations set up by Iranian Red Crescent Society have been available for 17 d with the aim of identifying and treating people with coronavirus, reducing road trips, and sensitizing people to the problem. This study aims to investigate the challenges of the procedure.

**Methods:**

A qualitative study was used to find the challenges of the COVID-19 screening centers. Volunteers, branch managers, and headquarter managers of the Iranian Red Crescent Society participated in this study applying snowball sampling. Data were collected by means of in-depth semi-structured telephone interviews in April 2020 after completion of the fever screening plan. All interviews were recorded and transcribed verbatim, always with prior permission of interviewees.

**Results:**

The interviews with 20 participants in the plan indicated 6 relevant challenges, including logistics, lack of planning, lack of coordination, legal challenges, mental health, and ethical challenges.

**Conclusions:**

The results indicated that, although establishing fever detection centers in Iran was a rapid response to COVID-19, it had significant flaws in the structure and adversely affected volunteers’ and staff’s health and financial resources. Therefore, well-structured protocols are required for similar responses in the future.

Coronavirus disease 2019 (COVID-19) pandemic is 1 of the worst disasters in the world since World War II.^1^ Since the identification of the first reported cases in China, the novel coronavirus needed only 3 mo to spread world-wide. Iran is among the top 20 countries in terms of infected people.^2^

Response to epidemics is different from other natural disasters, such as earthquakes and floods. As a result, the challenges in the course of humanitarian response are different. Most studies on response challenges deal with disasters triggered by geophysical hazards, hydrological hazards, or conflicts. There are few studies on the challenges of response to biological hazards, such as pandemics.

It could be due to the small share of pandemics compared with other disasters. Another reason might be different methods adopted to respond to epidemics. For research in epidemics, collaboration between public health professionals and social work professionals is vital.^3^ Some studies have mentioned the challenges of response to outbreaks (eg, Ebola, influenza, Zika virus, and the COVID-19 pandemic), such as aid workforce issues,^4^ personal protective equipment (PPE),^5^ data challenges,^6^ trust,^7,8^ ethical challenges,^9^ logistics,^10–12^ and psychosocial support. Response to epidemics comprised various dimensions, including aid workers, humanitarian organizations, and government.^13^ In addition, response is provided in different fields, including mental health response, health-care response, evacuation, quarantine, and burial. Determining the challenges is of vital importance because it will enhance the success rate in saving lives and alleviating hazards in the affected communities in the future.^14^

In Iran, one of response programs to COVID-19 was the project to launch the screening centers for travelers by Iranian Red Crescent Society (IRCS).^15^ That project required a vast capacity of financial resources, aid workers, and other logistics facilities. The screening program began on March 18, 2020, in several provinces and was extended within a week.^15^ In this plan, the IRCS operational forces worked in 851 temporary stations at the entry and exit gates of cities, train stations, and airports to control travelers and identify people suspected of having COVID-19. In each post, 4 trained aid workers (volunteers and staff) of the IRCS, screened the health of the travelers and referred the people in need of treatment to the health forces until April 4. Other major stakeholders of IRCS involved in this plan were the Ministry of Health, Judiciary, Police, and Ministry of Roads & Urban Development with the command of the governors in the provinces and the governors-general in the towns. During the implementation of the plan, totally 21,640,861 people were screened. The number of people screened and reported by the IRCS in the roads was estimated based on the number of passenger cars and 3 travelers per car. Screening was performed using thermometers.^16^ The IRCS said that 14,302 car travelers who were screened for fever had symptoms of coronavirus disease. Every day, 4930 volunteers and employees of the IRCS screening stations from different provinces were engaged in screening the travelers. A total of 95,371 aid workers implemented the plan using 15,278 vehicles per day. Stations were available 24 h a day.^16^

This study aims to find the challenges of this program from the perspective of aid workers and managers of IRCS.

## Methods

This study used qualitative study methods, including semi-structured interviews to investigate the challenges of a COVID-19 disease screening plan conducted in Yazd Province, Iran. In addition, we have applied a checklist for reporting qualitative research (SRQR).^17^ The inclusion criterion for the study was active participation in the fever screening plan as an aid worker or as a manager. A total of 24 aid workers and managers participated in the study. The sample comprised 7 branch managers, 8 managers of provincial headquarters, and 9 volunteer aid workers. The age of the participants ranged from 17 to 51 y. All interviews were recorded and transcribed verbatim, always with prior permission of the interviewees. The details of research methods are enclosed in the Supplementary File 1, including sampling strategy, ethical issues pertaining to human subjects, data collection methods, data collection instruments and strategies, units of study, data processing, data analysis, and techniques to enhance trustworthiness.

## Results

**Figure 1.**
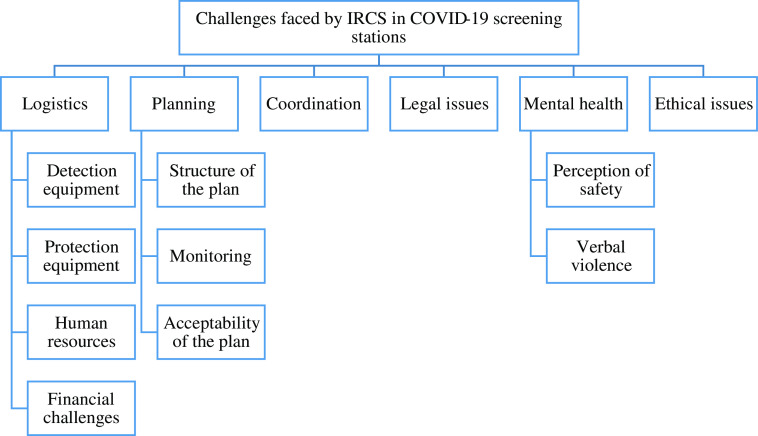
Challenges faced by Iranian Red Crescent Society in COVID-19 screening program of travelers.

Six items were found as main challenges faced by the IRCS in temperature screening stations: logistics, planning, coordination, legal issues, mental health, and ethical issues. A scheme of these challenges is illustrated in Figure 1. In addition, all interviews were coded and are shown in Supplementary File 2.

### Logistics

The major logistics problems identified in the study were as follows: (1) equipment for screening; (2) equipment for safety and protection; (3) human resources taking part in the plan; and (4) and financial challenges

One of the main problems was the troubles run into while using thermometers and pulse oximeters. Many devices either had problems from the first or broke down during operation, and it took time to replace them. On the other hand, lack of PPE, such as face shields, masks, protective cloths, and gloves, became one of the main problems during the plan, according to the interviews with all managers and aid workers (volunteers or staff). Recruiting too young volunteers (under 18 years) was a challenge facing the experienced aid workers, as well as a concern for their families. Due to shortage of time, some branches recruited less-trained aid workers, and it was obviously another issue in the program.

The money needed to buy items related to the plan was another challenge. Normally, a lengthy administrative process for spending is required within Red Crescent and other organizations in Iran. As the government allocates budget to the IRCS for disaster response, thus, according to law, the IRCS (as a nongovernmental organization) and governmental organizations are the same in provision and audit. Nevertheless, given the new circumstances, it was not possible to do so.

Another challenge was commitments of other organizations. The IRCS was only supposed to do screening, while other organizations would have to provide logistics and financial support. Nonfulfillment of commitments by other organizations was another issue causing logistics challenges in the program.

### Planning

Planning of the screening project was one of the challenges mentioned by the interviewees, shown in the Supplementary File 2. The project was launched shortly after the first case of coronavirus was identified in Iran. So, there was no prior preparation from the Red Crescent Society and other colleague organizations. Lack of structure for the plan (number of human resources and budget required for every station) was a challenge that branch managers were talking about it.

### Lack of Coordination

One of the other challenges indicated in interviews was lack of coordination. Weak internal coordination in the IRCS as well as poor external coordination between the IRCS and other governmental organizations was another issue mentioned by all interviewees. Although the level of coordination differed by city, there was a consensus about this issue as a challenge.

### Legal Challenges

Legal problems in the plan were among the challenges mentioned by branch managers. These problems are particularly acute when it comes to recruiting volunteers for the project. The volunteers were at risk of infection with coronavirus, and the branch managers did not know whether they would be legally responsible or not. Furthermore, legal considerations for formal expenditure were another challenge at the provincial level. On the 1 hand, expenditure has a long process in the IRCS, and on the other hand, suppliers (eg, mask suppliers) were not willing to go through this process because they had already enough customers. The financial manager and the logistics manager might have been held accountable if they made any unscheduled procurement beyond the approved process.

### Mental Health

The safety and security perception of aid workers and branch managers was one of the topics discussed in the interviews. Volunteers were psychologically stressed that the exposure and little protection while the activity might have cost them both their own lives and the lives of their families. Branch managers were also concerned about the health of the volunteers and were under psychological pressure. Verbal violence was another issue faced by aid workers in the plan. Travelers were frustrated by travel restrictions and long queues to cross the road, which led to verbal violence.

### Ethical Challenges

Ethical challenges in the project were among the issues posed by aid workers. For example, the volunteer worried that Red Crescent’s reputation would be jeopardized if he told the truth about the malfunction of the thermometer. Political issues are also a challenge for the plan. There was actually a pseudo-competition among governors in Yazd Province to work more and show more, which definitely put an excessive burden on RCS branches in counties. In addition, political pressure is against 2 humanitarian principles of IRCS, including independence and neutrality.

## Discussion

The results found by our study indicate that logistics planning, political challenges, lack of coordination, legal challenges, mental health, and ethical challenges were main challenges of IRCS in COVID-19 screening stations. This is in line with the results of other studies on humanitarian response challenges. Humanitarian organizations, such as Red Cross and Red Crescent Societies, have some common characteristics, including working under tremendous time pressure; engaging in complex stakeholder environments; budget dependency on media, wealthy, and goodwill donors; and relying on the sacrifices of dedicated humanitarian aid workers.^18,19^

Logistics is a significant challenge according to the findings of this study. Van Wassenhove found that approximately 80% of disaster relief efforts are related to logistics. It is shown that, to carry out an efficient program, an efficient logistics operation and supply chain management is needed.^20^

Coordination was another challenge that was mentioned in the interviews. Coordination is a set of techniques used to manage interactions within and between organizations.^21^ There are many stakeholders taking part in humanitarian responses, such as donors, government, philanthropic, business, militaries, affected people, humanitarian organizations, and civil society institutions.^13,22^ A necessary condition to have successful emergency operations is coordination.^21^ Coordination of funds, information, goods, services, equipment, and vehicles to avoid gaps and overlaps is very challenging.^23,24^

Perceptions of risk and insecurity by aid workers, on the 1 hand, and their feeling of having been neglected by managers on the other hand are significant challenges in the plan. In the risk sciences, the mentioned perceptions are called safety climate perception (physical and psychosocial). There are scientific evidences regarding many of the consequences of work stress, such as detriment to worker’s health, detriment to the quality of services and products, accidents, injuries, conflicts within the family and at the workplace, family breakdown, medications, and suicide.^25,26^

Haggman et al. investigated the mental health and occupational health of aid workers during an Ebola outbreak. They proposed and emphasized several items as follows: to provide additional training to encounter special circumstances, to avoid recruiting hesitant volunteers during an outbreak mission, the importance of peer support, the need of providing the health and welfare service for humanitarian aid workers in long-term and even after the mission, and to work in pairs.^27^ In a study about the mental health of aid workers during an Ebola outbreak humanitarian response, it was shown that risk perception has a vital role in willingness to respond to emergencies. It was indicated that aid workers had less tendency to participate in outbreak responses in comparison to other natural disasters, such as earthquakes and floods, for the fear of getting infected or transmitting infection to their families.^28,29^

There are similar findings on unwillingness to participate in humanitarian responses to pandemics such as the one that happened during the influenza outbreak in the United States in 2005.^30,31^ A study by Gershon et al.^32^ showed that aid worker responses to the Ebola outbreak were motivated by their beliefs, their sense of being useful to people, and a moral obligation. However, they had serious concerns about their own and their families’ health. It was investigated that several variables played a role in shifting the harm perspective held by the health workers. Institutional trust works as a key risk preventing factor, which is associated with previous experience, self-efficacy, care obligations, as well as humanitarian, ethics, and cognitive heuristics. Risk experiences may be exacerbated by any diagnosis of infection among coworkers or risk beliefs of family members and the public.^29^

The pressure put on humanitarian emergencies by politicians is a concern across the world. Many donor countries link their contribution to their political issues. This goes against humanitarian principles, including impartiality, neutrality, and independence.^18^

### Conclusions

Our study investigated the challenges faced by fever detection stations for COVID-19 in Iran. The IRCS showed its capacity for mobilizing a considerable number of volunteers in a short time. National plans require other factors before the launch to be successful, including detailed planning and determining its dimensions (financial, human, and logistically), proper distribution of items required in the plan, proper distribution of services, and identifying the impact of socio-political conditions on the implementation of the plan. The IRCS should also consider the psychosocial effects of such activities on its volunteers and staff in future programs and provide them with psychosocial support before initiating the plans. Providing a psychological profile for volunteers and selecting volunteers for each program according to their mental state are among the prerequisites that can help IRCS to carry out effective humanitarian programs in the future.

## Supplementary Materials

To view supplementary material for this article, please visit https://doi.org/10.1017/dmp.2021.219.
